# APPROACHES FOR INCORPORATING OLDER ADULT PERSPECTIVES IN ELDER MISTREATMENT PREVENTION

**DOI:** 10.1093/geroni/igae098.0143

**Published:** 2024-12-31

**Authors:** Ruthann Froberg, Randi Campetti

**Affiliations:** Virginia Tech, Waltham, Massachusetts, United States; Education Development Center, Waltham, Massachusetts, United States

## Abstract

This presentation discusses an approach for engaging older adult feedback on elder mistreatment (EM). EM can be a challenging topic to engage communities with due to the stigma and the sense of futility around solving this social issue. Despite these obstacles, a local community coalition to address EM successfully elicited feedback from older adults and service providers to create recommendations for their ongoing work. Collaboratively, member organizations developed three focus groups composed of participants aged 60 and above who were familiar with existing community resources for older adults. Member organizations also identified service providers to participate in an additional focus group in order to improve understanding of the services available to older adults. Interviewers conducted hour-long sessions with each group to gather feedback and insights on EM and related issues. Participants highlighted the importance of community engagement, addressing isolation, increasing digital literacy, and improving access to social services. Healthcare providers were recognized for their role in detecting EM, but challenges in accessing services persist. Service providers underscored the importance of collaboration and addressing barriers such as staffing and funding. The resulting recommendations, including accessible healthcare services, digital literacy initiatives, and integrated community care, reflect practical feedback to guide the coalition’s endeavors. This feedback has spurred renewed commitment within the coalition to address these recommendations in their ongoing work, highlighting the actionable insights garnered through community engagement.

